# Active Immunity Induced by Passive IgG Post-Exposure Protection against Ricin

**DOI:** 10.3390/toxins6010380

**Published:** 2014-01-21

**Authors:** Charles Chen Hu, Junfei Yin, Damon Chau, John W. Cherwonogrodzky, Wei-Gang Hu

**Affiliations:** Defence Research and Development Canada-Suffield, Box 4000, Station Main, Medicine Hat, AB T1A 8K6, Canada; E-Mails: c.hu@mail.utoronto.ca (C.C.H.); jfyin78@gmail.com (J.Y.); damon.chau@drdc-rddc.gc.ca (D.C.); john.cherwonogrodzky@drdc-rddc.gc.ca (J.W.C.); wei-gang.hu@drdc-rddc.gc.ca (W.-G.H.)

**Keywords:** passive antibody therapy, ricin intoxication, post-exposure, IgG, F(ab’)2, active immunity

## Abstract

Therapeutic antibodies can confer an instant protection against biothreat agents when administered. In this study, intact IgG and F(ab’)2 from goat anti-ricin hyperimmune sera were compared for the protection against lethal ricin mediated intoxication. Similar ricin-binding affinities and neutralizing activities *in vitro* were observed between IgG and F(ab’)2 when compared at the same molar concentration. In a murine ricin intoxication model, both IgG and F(ab’)2 could rescue 100% of the mice by one dose (3 nmol) administration of antibodies 1 hour after 5 × LD50 ricin challenge. Nine days later, when the rescued mice received a second ricin challenge (5 × LD50), only the IgG-treated mice survived; the F(ab’)2-treated mice did not. The experimental design excluded the possibility of residual goat IgG responsible for the protection against the second ricin challenge. Results confirmed that the active immunity against ricin in mice was induced quickly following the passive delivery of a single dose of goat IgG post-exposure. Furthermore, it was demonstrated that the induced active immunity against ricin in mice lasted at least 5 months. Therefore, passive IgG therapy not only provides immediate protection to the victim after ricin exposure, but also elicits an active immunity against ricin that subsequently results in long term protection.

## 1. Introduction

Vaccination can reduce the susceptibility of a population against specific biothreats provided that a safe vaccine exists. However, should a biothreat incident occur, the induction of a protective response by vaccination will likely take longer than the time between exposure and onset of the disease. This drawback of vaccines would limit their usefulness during an emergency response to a biothreat crisis. Therefore, vaccination is not appropriate for post-exposure medical countermeasures against biothreat agents. Also, it is controversial to vaccinate a large number of people for pre-exposure prophylaxis against an uncertain and unpredictable biothreat attack [1]. 

Unlike vaccines, therapeutic antibodies can confer an instant protection against biothreat agents when administered. Therapeutic antibodies can be administered in higher levels than those elicited in the recipient given vaccines, and would provide a high level of protection or treatment against a biothreat attack at a higher level than found in nature. Therapeutic antibodies are ideal for post-exposure protections. This passive antibody therapy has been shown to be effective against biothreat agents such as anthrax [2] and others [3,4,5]. 

Several approaches are available for the production of therapeutic antibodies. Human therapeutic antibodies are desirable and can be produced from the pooling of immune sera of vaccinated human volunteers. Unfortunately, limited sources prevent their widespread use as therapeutics. Therapeutic human or humanized monoclonal antibodies can also be created, but the slow and costly process of development has made a very few available for biodefence. Currently only one human monoclonal antibody has been approved by US Food and Drug Administration [6]. Animal vaccination has successfully been used to generate therapeutic antibodies specific for infectious and toxic agents such as snake venom [7,8], botulism toxin [9], and Ebola virus [10], but safety concerns have limited their widespread use in humans. Due to their foreignness to humans, multiple uses of animal antibodies will elicit immune responses (hypersensitivity reactions) in recipients [11], resulting in rapid clearance of the animal antibodies and anaphylaxis, the latter can sometimes be fatal. Although the most useful, human therapeutic antibodies against biothreat agents are currently not and will not be available any time soon even though the chance of a biothreat attack is now more likely than ever. As a backup plan, therapeutic antibodies from animals meet the urgent need for medical countermeasures against potential biothreats of mass destruction. These can be produced following the immunization of animals using inactive forms of biothreat agents. High yields of neutralizing antibodies can be generated rapidly in large animals (e.g., horse/sheep/goats) and then the hyperimmune sera can be processed quickly to make a product suitable for use in humans. 

Although intact antibodies are most commonly applied as therapeutics, these may not necessarily be the most efficient or desired molecular form depending on the application. Because of their large size (150 kD), intact antibodies tend to have slow tissue penetration speed [12]. In contrast, small antibody fragments, such as the fragment of antigen-binding of antibodies (Fab) and F(ab’)2 might distribute more rapidly throughout the tissues to neutralize toxins or infectious agents than intact IgG molecule. Fab is generated by papain digestion of intact antibodies to remove the entire fragment of crystallizable region (Fc), including the hinge region and then Fab is monovalent, containing only a single antigen binding site. The average molecular weight of Fab is about 50 kDa. The F(ab’)2 is generated by pepsin digestion of intact antibodies to remove most of the Fc but retain the hinge region. Therefore, F(ab’)2 has two Fab portions linked together by disulfide bonds and is divalent with the average molecular weight of around 110 kDa. These animal antibody fragments might also elicit less hypersensitivity reaction in humans than intact IgG due to their small size. However, Fab being so small is subject to being removed from the kidneys quickly, resulting in a very short half-life as compared to IgG and F(ab’)2 [13]. Furthermore, Fab’s monovalency results in the loss of functional affinity and reduced biological activity [14]. These disadvantages limit Fab applications as medical countermeasures against intoxication and infectious diseases. Therefore, most studies have been focused on the development of animal F(ab’)2 for applications in humans against rattlesnake bites [15], bee stings [16] and scorpion stings [17]. 

In the presented study, intact IgG and F(ab’)2 from goat anti-ricin hyperimmune sera were compared for protection against lethal ricin mediated intoxication. The results showed that the ricin-binding and neutralizing capabilities of goat IgG did not change significantly *in vitro* after removal of the Fc by pepsin digestion. It was also found that IgG and F(ab’)2 could protect mice against lethal ricin challenge when administered post-exposure. However, the protection in the murine model suggested a requirement for the Fc of the antibody to elicit subsequent active immune responses against ricin in mice. The anti-ricin protection provided by this active immunity occurred as early as 9 days after passive IgG administration post-exposure and lasted at least 5 months. 

## 2. Results and Discussion

Ricin is a 60–65 kDa glycoprotein derived from beans of the castor plant [18]. It consists of a ricin toxin A (RTA) protein and a ricin toxin B (RTB) protein linked by a disulfide bond. RTB binds to galactose residues on the mammalian cell surfaces to trigger cellular uptake of ricin. RTA enzymatically cleaves ribosomal RNA to stop protein synthesis [19]. Ricin is a highly potent toxin for humans [20]. Currently, there are no any vaccines and antidotes available against ricin. Mounting evidence has shown that antibodies against either subunit can neutralize ricin [21,22,23,24,25,26,27,28]. This study was designed to compare the anti-ricin properties of goat IgG and F(ab’)2 both *in vitro* and *in vivo* and assess their potentials for therapeutic applications.

### 2.1. Goat IgG and F(ab’)2 Preparation

**Figure 1:**
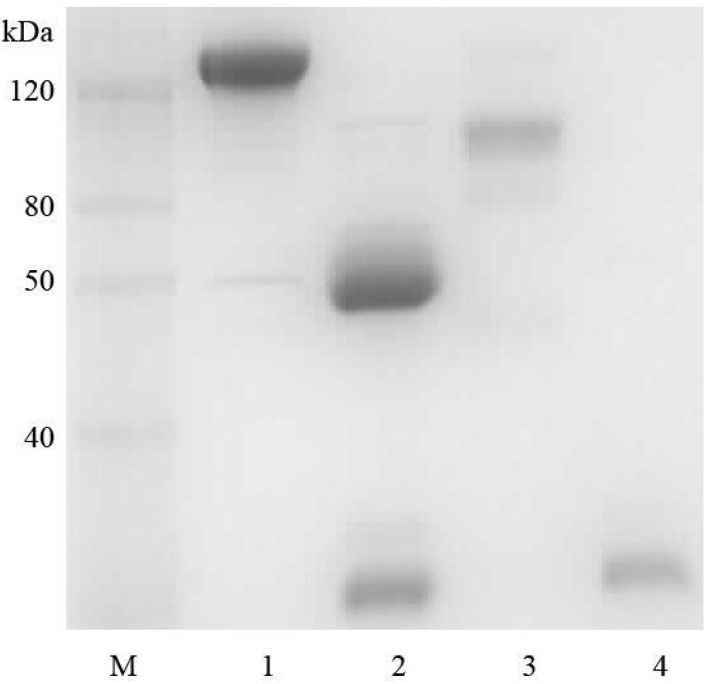
Sodium Dodecyl Sulfate Polyacrylamide Gel Electrophoresis (SDS-PAGE) analysis of goat F(ab’)2. Lane M is a molecular marker. Lanes 1 and 2 are goat IgG in non-reducing and reducing conditions. Lanes 3 and 4 are F(ab’)2 in non-reducing and reducing conditions.

Polyclonal IgG was purified from goat anti-ricin hyperimmune sera using protein G column. F(ab’)2 was prepared from goat IgG by pepsin digestion. To determine the optimal (digestion) cleavage time, immobilized pepsin was added to 250 µg goat IgG and incubated at 37 °C. Aliquots were removed at 1, 2, 4, 6, 8, and 18 h. As determined by Sodium Dodecyl Sulfate Polyacrylamide Gel Electrophoresis (SDS-PAGE), near complete pepsin digestion of IgG to F(ab’)2 appeared to occur after 18 h. The Fc was removed by passing the pepsin-treated IgG solution over a protein G column that allowed the F(ab’)2 to pass through. Following buffer exchange to phosphate-buffered saline (PBS) and subsequent concentration of the F(ab’)2 preparation using Amicon Centriprep devises, the final product was again analyzed by SDS-PAGE. As expected, only one major band was visualized; corresponding to a molecular weight (M) of ~110 kDa under non-reducing condition, and ~25 kDa under reducing condition (Figure 1). The purity was estimated at 90%. 

### 2.2. Affinity Assay for Goat IgG and F(ab’)2

**Figure 2:**
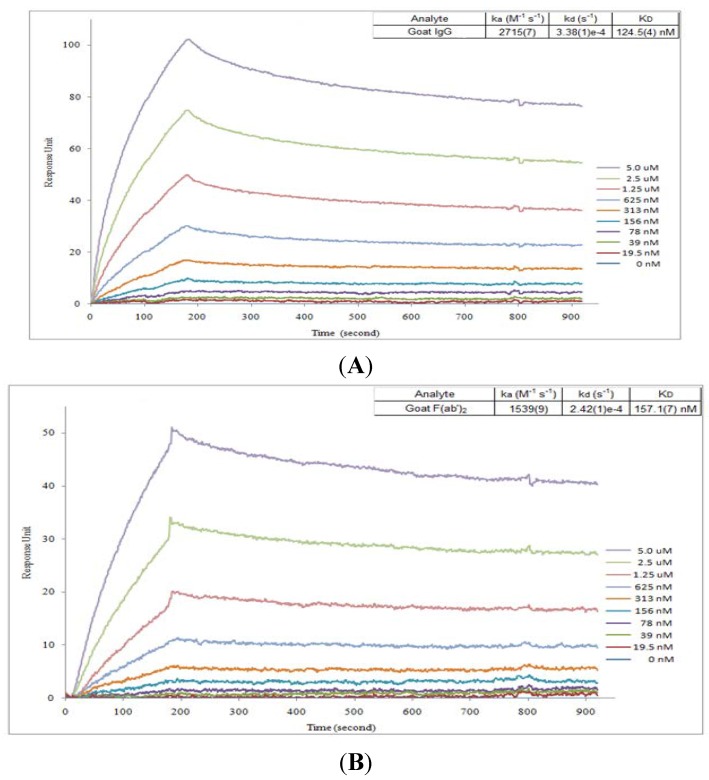
*In vitro* binding affinity analysis for goat IgG and F(ab’)2 by Surface Plasmon Resonance (SPR). SPR sensorgram of the kinetics of association and dissociation of a range of concentrations from 0 to 5 µM of goat IgG (A) or F(ab’)2 (B) to immobilized ricin.

In order to compare the ricin-binding affinity between IgG and F(ab’)2, measurements of the affinity constant (*K_D_*) for antibodies binding to ricin were performed by Surface Plasmon Resonance (SPR) biosensor. Ricin was captured on a biosensor chip, various concentrations of IgG or F(ab’)2 were passed through the chip. The kinetics of association and dissociation were recorded in a SPR sensorgram (Figure 2). The kinetic association and dissociation rate constants (*kon* and *koff)* were calculated from the ascending rate of resonance units during association and the descending rate during dissociation. The *K_D_* of IgG or F(ab’)2 to ricin was determined from the ratio of *koff/kon*. As shown in Figure 2, both IgG or F(ab’)2 had very similar affinity to ricin with *K_D_* of 124 nM or 157 nM.

### 2.3. Neutralization Assay for Goat IgG and F(ab’)2 *in Vitro*

**Figure 3:**
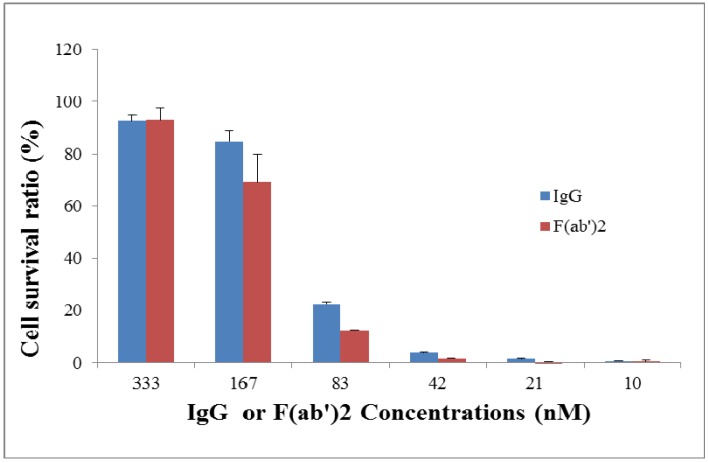
*In vitro* neutralization assay for goat IgG and F(ab’)2. Ricin (7.5 ng/mL) was pre-incubated with a serial dilution of goat IgG or F(ab’)2 for 2 h and then exposed to 10^4^ Vero cells/well for 2 days before evaluation of cell viability using Alamar blue staining.

Neutralizing capacities between IgG and F(ab’)2 were compared using a Vero cell toxicity neutralization assay with Alamar blue as an indicator. Ricin (7.5 ng/mL, 10 × LD50) was incubated with a serial dilution of IgG or F(ab’)2 (from 10 nM to 333 nM at two-fold dilution) for 2 h and then 10^4^ Vero cells/well were added into the mixture. After culture for 2 days, Alamar blue was added to evaluate viability of the Vero cells. As seen in Figure 3, cell survival ratios between IgG and F(ab’)2 were similar, not significantly different.

### 2.4. Efficacy of Goat IgG and F(ab’)2 *in Vivo*

**Figure 4:**
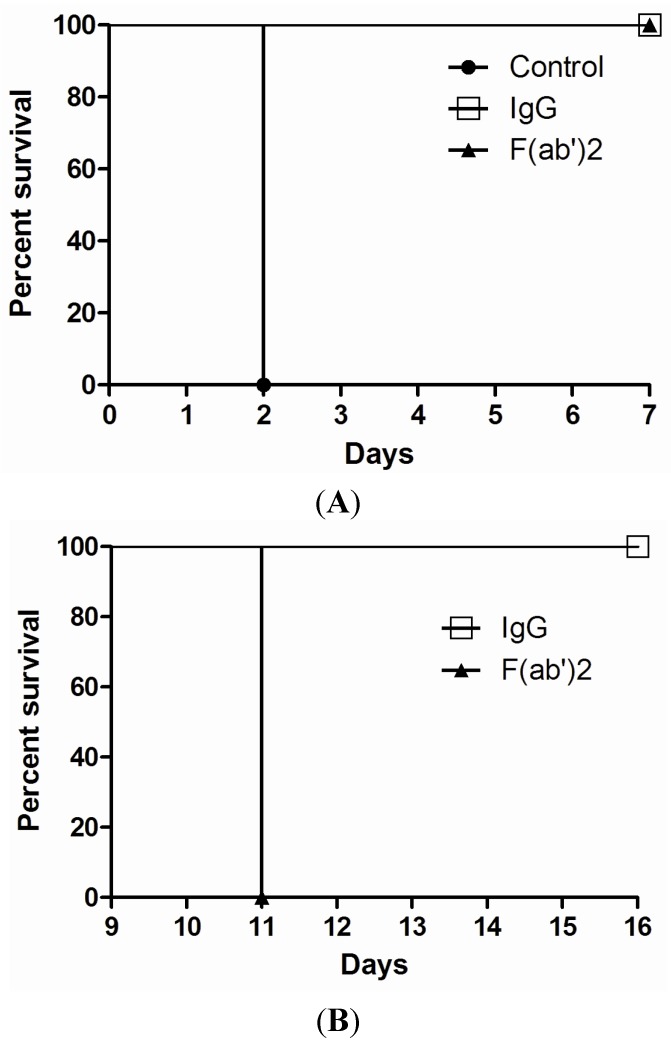
*In vivo* post-exposure protection assay for goat IgG and F(ab’)2. Ricin was given at the dose of 5 × LD50 to mice by i.p route. Goat IgG or F(ab’)2 at one dose of 3 nmol was administered i.p. at 1 h after ricin challenge and then mouse survival rate was monitored for 7 days (**A**); At day 9, mice were challenged with a second dose of 5 × LD50 of ricin and observed for 7 days (**B**). The experiment was repeated three times.

A mouse model was applied in order to evaluate the protective efficacy of goat IgG and F(ab’)2 in a post-exposure setting. Ricin was given at the dose of 5 × LD50 to mice by the intraperitoneal (i.p) route. As shown in Figure 4A, both goat IgG or F(ab’)2 rescued 100% of the mice by a single dose antibody injection (3 nmol) at 1 h post-challenge. All control mice died at day 2 post-challenge. Furthermore, after 9 days, surviving mice were challenged with a second dose of ricin (5 × LD50). All of the mice rescued by IgG were still protected without any death observed, while the mice rescued by F(ab’)2 died within 2 days of the second ricin challenge (Figure 4B). When the surviving mice were challenged again with 5 × LD50 of ricin after 5 months, these mice still survived (data not shown). However, if the dose of IgG or F(ab’)2 was reduced to 1.5 nmol at 1 h post-challenge, the mice were not rescued but the time of death of mice was extended up to 7–9 days post-challenge. Again, if the time of administration of IgG or F(ab’)2 (3 nmol) was delayed to 2 h post-challenge, not 100% of the mice were rescued. 

In 1992, Lemley, *et al*. reported that passive administration of a protective anti-ricin monoclonal antibody protected mice from not only an immediate challenge of ricin, but also a second challenge at 21 or 35 days after. The mice were actively immunized after passive antibody administration by pre-exposure prophylaxis against ricin challenge [28]. However, this group found that the induction of active immunity required monoclonal antibody and no anti-ricin active immunity was elicited when a goat polyclonal antibody was used. This result differs from that now presented here. The discrepancy may have resulted from the different dosages of goat polyclonal antibodies used in the two studies. They used 100 µg per mouse, while we used 450 µg (3 nM) per mouse. In our pilot studies, the dosage of less than 450 µg/mouse did not show any induction of active immunity. 

### 2.5. Anti-Ricin Antibody Levels in Mice

**Figure 5:**
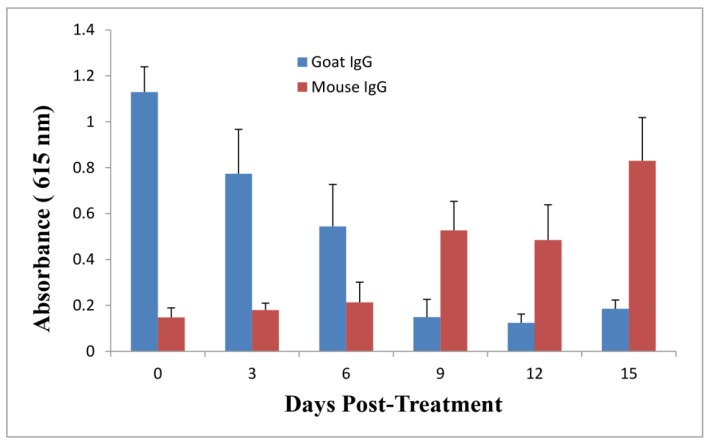
Evaluation of goat IgG and mouse IgG levels against ricin in pooled mouse sera over time by immunoassay. Goat IgG at the dose of 3 nmol was administered by the i.p. route into mice at 1 h post-challenge. Sera were collected at different time points for immunoassay to examine anti-ricin goat IgG and mouse IgG levels. All data represent the average of three independent experiments.

**Figure 6:**
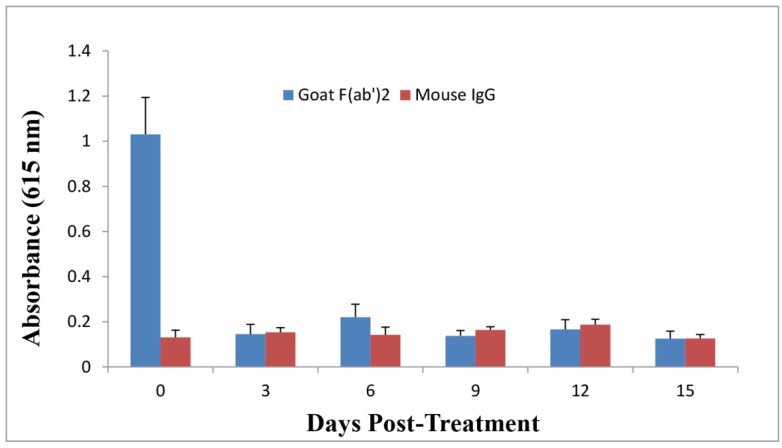
Evaluation of goat F(ab’)2 and mouse IgG levels against ricin in pooled mouse sera over time by immunoassay. Goat F(ab’)2 at the dose of 3 nmol was administered by the i.p. route into mice at 1 h post-challenge. Sera were collected at different time points for immunoassay to examine anti-ricin goat F(ab’)2 and mouse IgG levels. All data represent the average of three independent experiments.

F(ab’)2 has a shorter half life in serum as compared with IgG due to its smaller molecular weight without the Fc [13]. In order to investigate whether the second protection in goat IgG-treated mice resulted from residual IgG in mouse serum due to its longer half life than F(ab’)2, mouse sera were collected over time and an Enzyme-Linked Immunosorbent Assay (ELISA) was performed to monitor the levels of passive goat IgG, F(ab’)2, and active mouse IgG against ricin in serum after goat IgG or F(ab’)2 at the dose of 3 nmol was administered into mice at 1 h following a single dose of ricin challenge. As shown in Figure 5, the goat IgG level in mouse serum declined over time, returning to the base level after 9 days. Meanwhile, anti-ricin mouse IgG level increased over time to a considerable level at day 9, indicating that an active mouse anti-ricin immunity was elicited quickly following one dose of passive delivery of goat IgG after ricin challenge. In contrast, goat F(ab’)2 titer declined faster as compared to IgG, mouse IgG titer remained very low through the experiment (Figure 6). 

The only difference between IgG and F(ab’)2 is the Fc. Therefore, the Fc might play a pivotal role in the active immunity elicited quickly by the passive delivery of goat IgG post-exposure therapy against ricin. 

Antigen presentation to CD4 T cells by professional antigen-presenting cells (APC) is a critical event in the generation of protective humoral immunity. APC, including dendritic cells, macrophages, and B-cells need to capture and internalize antigens either by phagocytosis or by receptor-mediated endocytosis before these can process the antigens to display antigen fragments, on class II major histocompatibility complex molecules on their surface, to CD4 T cells [29]. In return, the activated CD4 T cells help to activate B-cells for production of antigen specific antibodies. All of the professional APC have Fc receptors (FcR) on their surface. Numerous studies have demonstrated that targeting antigens to FcR on APC can significantly enhance antigens specific immunity [30,31,32]. IgG, when binding to antigens, is capable of mediating FcR-dependent antigen presentation, and thereby enhance CD4 T-cell activation by 100 to 1000 fold [31,33]. Furthermore, when targeting antigens to FcR, adjuvant is not required, and immunity can be dramatically enhanced [30,32]. As an example, in the absence of alum, tetanus toxoid (TT)-Fc fusion protein was superior to the commercial vaccine (TT plus alum) at inducing TT-specific antibodies *in vivo* [34]. 

In this study, when goat IgG bound to ricin, the Fc of goat IgG might cross-react with mouse FcR on APC to enhance APC internalization of ricin, helping antigen presentation and thereby dramatically eliciting active anti-ricin immunity. The internalized ricin might not exert toxicity within APC due to the IgG blockage of ricin. Unlike IgG, F(ab’)2 could not help antigen presentation due to its lack of the Fc and so failed to elicit any rapid active immunity against ricin even though F(ab’)2 was comparable to IgG in terms of post-exposure protection against ricin intoxication. If long term protection against a ricin is required, F(ab’)2 has to be repeatedly administered into the host. However, there is evidence showing that the repeated delivery of F(ab’)2 to different species would elicit a humoral response against the F(ab’)2 despite its relatively smaller molecule as compared to IgG [35]. As a matter of fact, when antibodies were administered by the intravenous route, there was no significant difference in immunogenicity between IgG and F(ab’)2 [36], while passive antibody therapies are generally administered intravenously due to a large of volume required. When human anti-animal F(ab’)2 antibody response is developed due to the repeated delivery of F(ab’)2, it will clear F(ab’)2 from humans quickly, resulting in dramatic decrease of F(ab’)2 efficacy and possible hypersensitive responses with horrific consequences. In contrast to this possibility and as a likely outcome of results now presented, one dose of IgG could not only rescue the victim after a ricin crisis, but also quickly elicit the active immunity for long term protection while reducing the potential of anaphylactic anti-animal immune responses, resulting from the repeated delivery of animal antibodies. 

The administration of antibodies at 1 h post-exposure in this study is not practical in a ricin crisis. However, it should be taken into account that the delivery route of ricin in this study was i.p. Realistically, ricin would most likely be used by terrorists through an aerosol. The time course of ricin intoxication is dependent on the delivery route of ricin. For example, 5 × LD50 ricin administered i.p. led to the death of mice within 1–2 days [37,38], while the same dose of ricin administered intranasally would cause the death of mice within around 4 days [21]. In a lung-challenge model, it was found that when polyclonal antibodies were administered up to 18 h after ricin poisoning, all animals survived [39], while in a peritoneal cavity-challenge model, the rescuing polyclonal antibodies had to be administered within 1.5 h following ricin challenge in order to rescue the mice [37]. Therefore, it is probable that the administration of anti-ricin antibodies to the victim at several or a dozen hours post-exposure to aerosolized ricin might still be able to provide immediate protection and elicit subsequent active immunity for long term protection against ricin poisoning. 

## 3. Experimental Section

### 3.1. Preparation of Goat IgG and F(ab’)2

Goat anti-ricin hyperimmune sera were prepared by Cangene Corporation (Winnipeg, MB, Canada), which contracted Capralogics Inc. (Gilbertville, MA, USA) to immunize US Department of Agriculture-certified scrapie-free goats with Twinstrand Therapeutics Inc.’s ricin toxoid. 

Polyclonal IgG was purified from the goat anti-ricin hyperimmune sera using a protein-G purification kit (Fisher Scientific, Ottawa, ON, Canada) following the manufacture’s instruction. Purified IgG was supplied at a concentration of 15 mg/mL.

F(ab’)2 was prepared using a F(ab’)2 preparation kit (Fisher Scientific) according to the manufacturer’s instructions. To determine the extent of digestion and confirm the presence and purity of F(ab’)2, 10% SDS-PAGE gels were run under reducing and non-reducing conditions. SDS-PAGE gels were visualized by SimplyBlue Safestain staining (Life Technologies Inc., Burlington, ON, Canada). The molecular weights of samples were estimated by comparison to the relative mobility values of standards of known molecular weight. The image of the stained SDS-PAGE gel was recorded using the VersaDoc 5000MP imaging system (Bio-Rad Laboratories, Mississauga, ON, Canada). The F(ab’)2 was dialyzed extensively against PBS and then concentrated by Amicon Centriprep-50 devices (Billerica, MA, USA). The final product was stored at −20 °C until use.

### 3.2. Preparation of Ricin Stock

Ricin was prepared from castor bean seeds in Defence Research and Development Canada (DRDC)-Suffield. The toxicity of ricin stock was also determined. One LD50 of ricin for mice was determined by i.p. injection of a series of ricin dilution into mice. The mice were observed for 7 days. The amount of ricin for 1 × LD50 delivered by the i.p. route for one mouse was 0.215 µg; 5 × LD50 was 1.075 µg, or about 1 µg. For 5 × LD50 of ricin delivered by the i.p. route, mice died within 2 days. All research with ricin was conducted in a secure biosafety level 2 area and under the preview of the National Authority (Department of Foreign Affairs and International Trade-Ottawa) for the Organization for the Prohibition of Chemical Weapons.

### 3.3. Affinity Analysis

The affinities for goat IgG and F(ab’)2 binding to ricin were determined using a SPR biosensor, SensiQ Pioneer (ICx Technologies, Oklahoma, OK, USA). Briefly, ricin (10 μg/mL) diluted in 10 mM acetate buffer pH 4.5 was first immobilized onto the COOH1 chip following the standard 1-ethyl-3-(3-dimethylpropyl)-carbodiimide plus *N*-hydroxysuccinimide (Sigma-Aldrich, Oakville, ON, Canada) coupling chemistry and 250 response units of immobilized ricin. The system was operated at 25 °C. Kinetic measurements were carried out by 2 min injection at a flow rate of 25 μL/min of serial dilutions of IgG or F(ab’)2 up to 5 µM in 4-(2-hydroxyethyl)-1-piperazineethanesulfonic acid-buffered saline containing 3 mM ethylenediaminetetraacetic acid, 150 mM NaCl and 0.005% Tween-20 and dissociation for 6 min. The ricin immobilized chip surface was regenerated by injection of 10 mM phosphoric acid for 120 s after each cycle. The data of *koff* and *kon* rate constants were obtained with the SensiQ Qdat software, corrected by subtraction of the zero antibody concentration flow cell as well as the zero ricin flow cell. The *K_D_* values were calculated from the ratio of *koff* and *kon*.

### 3.4. *In Vitro* Neutralization Assay

A Vero cell (ATCC, Burlington, ON, Canada) toxicity neutralization assay with Alamar blue as an indicator was performed. Ricin was incubated with a serial dilution of IgG or F(ab’)2 for 2 h at 37 °C in 96-well plates. Ten thousand Vero cells cultured in 50 µL of Dulbecco’s modification of Eagle medium (Life Technologies Inc.) with 10% fetal bovine serum (Hyclone, Fisher Scientific) were added into the mixture. The final volume of the cell mixture was 200 µL with ricin concentration of 7.5 ng/mL (10 × LD50), IgG or F(ab’)2 concentrations from 10 to 333 nM. After incubation at 37 °C, 5% CO2 for 2 days, 20 µL of Alamar blue (TREK Diagnostic System, Cleveland, OH) was added to each well and the plate was further incubated for 6–7 h. On a plate reader (Molecular Devices, Sunnyvale, CA, USA), the plate was read at an absorbance of 570 nm with 600 nm as a reference wavelength. Readings were normalized by subtracting the absorbance reading of wells without cells. The cell viability was expressed as cell survival ratio relative to the control without ricin (Vero cells plus antibodies).

### 3.5. *In Vivo* Protection Assay

Female Balb/c mice (6 week old, 20–25 g) were obtained from the pathogen-free mouse-breeding colony at DRDC-Suffield, with the original breeding pairs purchased from Charles River Canada (St Constant, QC, Canada). 

All mouse experiments were performed in strict accordance with the guidelines set out by the Canadian Council on Animal Care. The animal care protocol was reviewed and approved by the Committee on the Ethics of Animal Experiments of DRDC-Suffield (protocol number: J1C-10-1-1-0). All efforts were made to minimize suffering to use alternative end points based on symptom assessment. A scoring symptom chart was applied in which 0 = no symptoms; 1 = ruffled fur; 2 = 1 + humped back and huddled; 3 = 2 + resistance but some mobility when prodded and eyes closed; 4 = 3 + a lack of response or mobility and reduced breathing. Mice were terminated when these reached a score of 4. If mice were controls and had not received therapy, these were unlikely to recover and so were terminated at a score of 3.

For the post-exposure therapeutic efficacy study, groups of 4–8 mice were given 5 × LD50 of ricin per mouse by the i.p. route and then 3 nmol of goat IgG or F(ab’)2 per mouse administered by the i.p. route at 1 h post-ricin challenge. After 9 days or 5 months, the surviving mice were challenged with 5 × LD50 again. The mice were observed for morbidity and mortality over one week after each ricin challenge.

### 3.6. ELISA

ELISA was performed to evaluate anti-ricin antibodies. Ninety-six-well Nunc Maxisorp ELISA plates (Canadian Life Technologies, Burlington, ON, Canada) were coated with 100 µL per well of 2.5 µg/mL ricin in carbonate bicarbonate buffer, pH 9.6, then incubated overnight at 4 °C. After blocking with SuperBlock blocking buffer (Fisher Scientific), the plates were incubated with 100 µL of serum dilutions for 2 h at room temperature. Anti-ricin antibodies were detected by incubation with 1:3000 diluted horseradish peroxidase-bovine anti-goat IgG (H + L) or goat anti-mouse IgG (H + L) (Jackson ImmunoResearch, West Grove, PA, USA) followed by the addition of tetramethylbenzidine (Kirkegaard and Perry Laboratories, Gathersburg, MD, USA). Absorbance was measured at 615 nm by a microplate autoreader (Molecular Devices).

## 4. Conclusions

Anti-ricin passive goat IgG antibody therapy might be an ideal approach to provide immediate protection to the victim exposed to ricin and has additional benefits of eliciting subsequent active anti-ricin immunity for long term protection against ricin poisoning. 
